# Supplementary material for: EnzML: multi-label prediction of enzyme classes using InterPro signatures
Source: BMC Bioinformatics. 2012 Apr 25;13:61. doi: 10.1186/1471-2105-13-61 (PMC3483700; doi:10.1186/1471-2105-13-61)
Supplement: Addtional file 5 — The Java code to format the data files, evaluate and predict. The file enzml_java_code.tar.gz contains the Java code used to format database data to ARFF and XML formats, to execute cross and train-test (jackknife) evaluations and to record evaluation results to database. More information is included in the readme.txt file and the Javadoc files. The code can be used with a MySQL database. To use a different database software, other JDBC drivers might be required. [file 1471-2105-13-61-S5.gz › java_code/utils/doc/uk/ac/ed/inf/utils/database/class-use/DbConn.html]

Uses of Class uk.ac.ed.inf.utils.database.DbConn


---


|  |  |  |
| --- | --- | --- |
| **Overview** \| **Package** \| **Class** \| **Use** \| **Tree** \| **Deprecated** \| **Index** \| **Help** | |  |
| PREV   NEXT | **FRAMES**    **NO FRAMES**     **All Classes** |  |


---


## **Uses of Class uk.ac.ed.inf.utils.database.DbConn**

| Packages that use DbConn | |
| --- | --- |
| **test.database** |  |
| **uk.ac.ed.inf.utils.database** |  |

Uses of DbConn in test.database

| Methods in test.database that return DbConn | |
| --- | --- |
| `static DbConn` | `DbConnPropsTest.getDbConnProps()` |

Uses of DbConn in uk.ac.ed.inf.utils.database

| Methods in uk.ac.ed.inf.utils.database that return DbConn | |
| --- | --- |
| `DbConn` | `DbManaged.getDbConnection()` |
| `DbConn` | `DbManager.getDbConnection()` |
| `DbConn` | `DbConn.getOpenConnection()` |

| Methods in uk.ac.ed.inf.utils.database with parameters of type DbConn | |
| --- | --- |
| `static boolean` | `DbUtils.elementExistsInTable(java.lang.String table, java.lang.String column, java.lang.String element, DbConn dbConn)`             Get if an element exists in a table column |
| `static boolean` | `DbUtils.elementsExistsInTable(java.lang.String table, java.lang.String column1, java.lang.String element1, java.lang.String column2, java.lang.String element2, DbConn dbConn)`             Get if two elements exists in the same table row |
| `static boolean` | `DbUtils.existsInTable(java.lang.String query, DbConn dbConn)` |
| `static java.sql.ResultSet` | `DbUtils.getResultSet(java.lang.String sql, DbConn dbConn)`             Executes an sql query returning a result set |
| `static boolean` | `DbUtils.insert(DbConn conn, java.lang.String insertSql)`             Executes sql insert and returns whether the insert was successful. |
| `static java.lang.Integer` | `DbUtils.insertReturnsKey(DbConn conn, java.lang.String insertSql)`             Executes sql insert and returns the auto generated key for the first inserted row. |
| `static boolean` | `DbUtils.similarElementExistsInTable(java.lang.String table, java.lang.String column, java.lang.String element, DbConn dbConn)` |

---


|  |  |  |
| --- | --- | --- |
| **Overview** \| **Package** \| **Class** \| **Use** \| **Tree** \| **Deprecated** \| **Index** \| **Help** | |  |
| PREV   NEXT | **FRAMES**    **NO FRAMES**     **All Classes** |  |


---
